# Therapeutic Advancements in Multiple Myeloma

**DOI:** 10.3389/fonc.2014.00241

**Published:** 2014-09-04

**Authors:** Alessandro Gozzetti, Veronica Candi, Giulia Papini, Monica Bocchia

**Affiliations:** ^1^Azienda Ospedaliera Universitaria Senese, Policlinico “Santa Maria alle Scotte”, Siena, Italy

**Keywords:** multiple myeloma, young, elderly, bortezomib, IMID’s, new therapies

## Abstract

Multiple myeloma survival has significantly improved in the latest years due to a broad spectrum of novel agents available for treatment. The introduction of thalidomide, bortezomib, and lenalidomide together with autologous stem-cell transplantation has considerably increased complete remission rate and progression-free survival resulting ultimately in prolonged survival in myeloma patients. Moreover, novel strategies of treatment such as consolidation and maintenance are being used to further implement responses. Finally, a number of new drugs such as carfilzomib and pomalidomide are already in clinical practice, making the future of myeloma patients brighter.

## Introduction

Multiple myeloma (MM) is a plasma cell malignancy characterized by clonal proliferation of plasma cells in the bone marrow microenvironment and associated organ damage (CRAB = increased calcium, renal insufficiency, anemia, bone lesions) (1). The organ damage is due to a monoclonal protein produced in the blood or urine. It represents about 10% of hematological cancers and 1% of all cancers. Median age at diagnosis is 70 years. In the last decades, we experienced a great improvement in myeloma survival both in young and old patients (2, 3). In fact, 5-year relative survival increased from 28.8 to 34.7% and 10-year relative survival increased from 11.1 to 17.4% between 1990–1992 and 2002–2004. A more evident increase was seen in the age group younger than 50 years, leading to 5- and 10-year relative survival of 56 and 41% in 2002–2004, and in the age group 50–59 years, leading to 5- and 10-year relative survival of 48 and 28% in 2002–2004. By contrast, only moderate improvement was seen in the age group 60–69 years, and no substantial improvement was achieved among older patients (3). The clinical progresses are related to the introduction of novel agents (bortezomib, thalidomide, lenalidomide) and especially for young patients, to autologous stem-cell transplantation (ASCT). These approaches resulted in an increased rate of complete response (CR) that translated into prolonged survival and improved quality of life. Additionally, peculiar extra medullary localizations of MM seemed to benefit from novel agents therapy (4). Nonetheless, a better understanding of plasma cell biology and myeloma pathways has resulted in the identification of novel targets for therapy. New agents deriving from already approved and active agents (such as second- and third generation proteasome inhibitors, thalidomide, lenalidomide) have been developed and also new drugs with novel mechanisms of action are under investigation in clinical trials.

Although advancements have been outstanding in the field of myeloma, there is still a small group of patients (10–15%) that has a dismal prognosis, i.e., del 17p and t(4;14) patients, in which novel therapeutic approaches are urgently warranted (5–7).

## Initial Treatment of Transplant Eligible Myeloma Patients

Today patients younger than 65 years are usually eligible for ASCT. As induction treatment, three or four cycles of therapy are usually given and less than six cycles are recommended (8).

Induction therapy is given to reduce tumor burden before stem-cell harvest, and drugs that can compromise hematopoietic stem-cell collection should be avoided (alkylating agents) (8).

On the basis of the available data from phase II studies, three-drug combination regimens are considered as the standard of care for use as induction therapy prior to ASCT. Thalidomide was the first novel agent compared with vincristine, adriamycin, dexamethasone (VAD) either in combination with dexamethasone or with dexamethasone and doxorubicin (TAD), the latter with a little benefit (9). Data of the most commonly used agents in phase III trials are summarized in Table 1. The most effective combinations include proteasome inhibitors plus either thalidomide, lenalidomide, or chemotherapy. Bortezomib in combination with dexamethasone (VD) was compared with VAD (10) with induction of CR/near-CR in 15 vs. 6% and overall response rates 79 vs. 63%, respectively. Even after ASCT, responses were confirmed superior for VD: CR/nCR: 35 vs. 18%. Median progression-free survivals (PFS) were 36 vs. 30 months with VD vs. VAD, respectively, yet survival that was not superior in the VD arms maybe due to effective salvage regimens at the time of relapse.

**Table 1:** **Results of phase III trials employing novel agents as induction therapy in young patients**.

Therapy	*N*	Reponse post-induction		Reponse post-ASCT		PFS (months)	OS
		CR/nCR (%)	>VGPR (%)	CR/nCR (%)	>VGPR (%)	
VD	121	15	38	35	68	36	NR (3 years)
VTD	241	31	63	71	89	NR	NR (3 years)
PAD	413	15	42	49	76	35	NR (5 years)
VTD	130	35	60	46	U	56	NR (4 years)

*U, unreported; NR, not reached*.

The combination of bortezomib–thalidomide–dexamethasone (VTD) has proved to be superior to thalidomide–dexamethasone (TD) as induction therapy before ASCT resulting in a 3-year PFS of 68% for the VTD arm vs. 56% for the TD arm (11). VTD was also superior in a Spanish study associated with thalidomide maintenance (12). The addiction of doxorubicin to bortezomib–dexamethasone (PAD) was superior to VAD followed by ASCT and thalidomide maintenance, with a median PFS of 35 vs. 28 months, respectively (13).

The association of bortezomib as induction therapy with other one or two drugs showed a superior efficacy in terms of responses, but none showed so far superiority in terms of overall survival (OS). Phase II trials have showed that the addition of cyclophosphamide (VCD) or lenalidomide (VRD) can be feasible with at least partial remission (PR) in 97 and 100% of patients, respectively (14, 15). In another phase II study, the four drugs combination with bortezomib–dexamethasone–cyclophosphamide–lenalidomide (VCDR) appeared to be a good induction option (16) with a CR rate of 25% and a very good partial response (VGPR) rate of at least 58%.

The novel proteasome inhibitor carfilzomib was also tested in phase II studies in newly diagnosed patients: in combination with lenalidomide and dexamethasone (CRd) showed outstanding responses with CR/nCR in 67% (17). In another study, Carfilzomib–thalidomide–dexamethasone was given as pre-transplant induction and post-transplant consolidation and led to 18% CR and 91% >PR (18).

### Consolidation/maintenance

Therapy consolidation (usually two cycles after ASCT to increase responses) and maintenance (continuous therapy until progression) are being explored to improve outcome after ASCT as an alternative to perform a second autotransplant with the idea to achieve the same efficacy but with less toxicity. In one study, VTD consolidation increased CR from 15 to 49% in patients who had previously achieved VGPR after double ASCT (19). Molecular remissions by allele-specific polymerase chain reaction (AS-PCR) following VTD treatment had a better outcome: the PFS at 42 months for patients with a low tumor load was 100 vs. 57% for patients with a higher tumor load after VTD. Another study confirmed these findings (20). Two cycles of consolidation therapy with TD or VTD were given after the second ASCT. In the TD arm, consolidation improved the CR rate from 40 to 47%. In the VTD arm, the CR rate increased from 49 to 61%. Several studies are ongoing to better evaluate the role of consolidation therapy.

Maintenance has been explored first with thalidomide and subsequently with lenalidomide (21–23). Six phase III studies have shown a benefit for thalidomide in terms of response and PFS, but OS was improved only in two of these trials (24–28). Yet, grade 3–4 polyneuropathy was a major concern (7–19%), inducing 52% of median discontinuation rate. Lenalidomide is currently considered as the best candidate for use as maintenance therapy because of a safer profile. Results from two randomized trials evaluating lenalidomide maintenance following ASCT have recently been published. Although an advantage in progression-free survival was seen in both trials, a benefit in OS for patients receiving maintenance therapy was observed only in the trial published by McCarthy et al. (23). However, this benefit was limited to those patients who had not achieved a CR on their previous treatment strategy. Questions have been raised about the need of a maintenance therapy for every myeloma patient (29, 30). In addition to carefully consider the risk–benefit ratio for a patient during maintenance therapy (increase of second malignancies), patient’s quality of life and treatment cost effectiveness should be also evaluated. These findings highlight the importance of identifying the optimal duration of therapy and risk factors for this complication.

## Initial Treatment of Non-Transplant Eligible Myeloma Patients

About two-thirds of MM patients are more than 65 years old at the time of the first diagnosis. Therefore, the majority of patients are usually not eligible for high-dose therapy followed by ASCT. Especially in the elderly, the treatment must be individualized because of their vulnerability that can complicate both the presentation and management of MM (31, 32). As such, it is mandatory to take into consideration the “biological” age of the patient not only the chronologic age, including the evaluation of both the performance status by score such as Karnofsky scale and the comorbidities by geriatric score (33). Age-related organ functions and metabolic changes can contribute to the poor tolerability of treatments as well as to increased treatment-related adverse events. Due to these toxicities, dose adjustments are often required with a consequent reduction of dose intensity that can lead to the poorer outcome observed frequently in elderly patients. The prolongation of PFS and OS must remain as initial aims in the management of old MM patient, although the quality of life should ultimately prevail in the oldest and fragile ones.

## Induction Therapy: What is the Best Treatment?

Achieving at least a VGPR has been demonstrated to be related to an improvement of the long-term outcome also in the elderly patients (34). Standard frontline treatment for elderly patients has been for long time the combination of the oral alkylating agent melphalan with prednisone (MP). This schedule is well tolerated even in frail patients and can be administered as outpatient regimen with maintenance of a good quality of life but the overall response rate obtained is dismal. The introduction of novel agents, such as thalidomide, lenalidomide, and bortezomib, has led to better responses also in this setting of patients. Since the CR is an independent predictor of longer PFS and OS regardless of age and International Staging System (ISS), a novel agent is recommended in the induction therapy.

Six randomized studies have compared the efficacy and safety of the standard MP regimen to the new combination of MP plus thalidomide (MPT) (35–41). These trials reported an evident improvement of the overall response rate and the PFS associated with the MPT regimen with respect to MP, but the advantage in OS is unclear. There are two meta-analyses of these data that confirmed a significant improvement in PFS (5.4 months) and a “trend” toward significant improvement in OS (6.6 months) when thalidomide is added to MP as a frontline treatment in elderly patients (42). In addition, the improvement seems to be less pronounced in patients aged 75 years and the optimal dose of thalidomide is not established as most of the clinicians adapted the dose according to patient’s status and occurrence of side effects or toxicity. At least 75% of the grade 3–4 toxicities occurred during the first 6 months of treatment. Neuropathy, deep-vein thrombosis, and dermatological toxicity were the most frequent thalidomide-related adverse events, while hematologic toxicities seemed to be related to melphalan doses.

Cyclophosphamide is another alkylating agent that can be associated with thalidomide and this association achieved an improvement in overall response rate compared to the standard MP (64 vs. 33% respectively), but in terms of PSF and OS there are no differences between the two regimens (43).

A randomized phase III study compared bortezomib–melphalan–prednisone (VMP) to MP also in the elderly. VMP significantly increased the CR rate (from 4 to 30%), PFS (from 16 to 23 months), and OS (from 43 to 56 months) with respect to MP (44). Subsequently, a reduced bortezomib schedule (from twice- to once-weekly administration) was shown to be better tolerated without affecting the outcome (45). Today, both MPT and VMP are considered the standard therapies for elderly patients (Table 2).

**Table 2:** **Main regimens used in elderly MM patients**.

Therapy	CR (%)	PFS (months)	OS
MPT	7–23	15–28	28–52 months
VMP	30	24	68% at 36 months
Rd	4	25	76% at 24 months
MPR-R	10	31	70% at 36 months
VMPT-VT	38	35	61% at 60 months

More recently, a phase III trial compared lenalidomide plus high-dose dexamethasone (RD) to lenalidomide plus low-dose dexamethasone (Rd) in newly diagnosed MM patients. Rd induced a significantly longer 1-year OS and a lower toxicity compared to RD (46). The three-drugs regimen melphalan–prednisone–lenalidomide followed by lenalidomide maintenance (MPR-R) was lately compared to MPR and MP in a phase III trial. In this study, MPR-R significantly reduced the risk of progression with respect to MPR and MP. The CR rate was 10% for MPR-R and 3% for both MPR and MP. MPR-R also significantly prolonged median PFS (31 vs. 14 vs. 13 months, respectively) (47).

Bortezomib plus thalidomide (VT) maintenance was assessed in two trials (48, 49). In both studies, PFS was improved although OS only in one.

## Conclusion

Many new treatments are now available for MM patients both transplant-related and non-transplant-related and a continuous improvement in disease free and OS is expected with the advent of the newest drugs. If prolongation of OS should be always the first aim of treatment in the management of elderly and fragile patients, quality of life should be carefully evaluated.

## Conflict of Interest Statement

The authors declare that the research was conducted in the absence of any commercial or financial relationships that could be construed as a potential conflict of interest.
